# A randomised controlled trial of six weeks of home enteral nutrition versus standard care after oesophagectomy or total gastrectomy for cancer: report on a pilot and feasibility study

**DOI:** 10.1186/s13063-015-1053-y

**Published:** 2015-11-21

**Authors:** David J. Bowrey, Melanie Baker, Vanessa Halliday, Anne L. Thomas, Ruth Pulikottil-Jacob, Karen Smith, Tom Morris, Arne Ring

**Affiliations:** Department of Surgery, University Hospitals of Leicester NHS Trust, Level 6 Balmoral Building, Leicester, UK; School of Health and Related Research, University of Sheffield, Sheffield, UK; Department of Cancer Studies, University of Leicester, Leicester, UK; Department of Health Economics, University of Warwick, Coventry, UK; Department of Health Sciences, University of Leicester, Leicester, UK; Leicester Clinical Trials Unit, University of Leicester, Leicester, UK; Current affiliation: Department of Mathematical Statistics and Actuarial Science, University of the Free State, Bloemfontein, South Africa

## Abstract

**Background:**

Poor nutrition in the first months after oesophago-gastric resection is a contributing factor to the reduced quality of life seen in these patients. The aim of this pilot and feasibility study was to ascertain the feasibility of conducting a multi-centre randomised controlled trial to evaluate routine home enteral nutrition in these patients.

**Methods:**

Patients undergoing oesophagectomy or total gastrectomy were randomised to either six weeks of home feeding through a jejunostomy (intervention), or treatment as usual (control). Intervention comprised overnight feeding, providing 50 % of energy and protein requirements, in addition to usual oral intake. Primary outcome measures were recruitment and retention rates at six weeks and six months. Nutritional intake, nutritional parameters, quality of life and healthcare costs were also collected. Interviews were conducted with a sample of participants, to ascertain patient and carer experiences.

**Results:**

Fifty-four of 112 (48 %) eligible patients participated in the study over the 20 months. Study retention at six weeks was 41/54 patients (76 %) and at six months was 36/54 (67 %). At six weeks, participants in the control group had lost on average 3.9 kg more than participants in the intervention group (95 % confidence interval [CI] 1.6 to 6.2). These differences remained evident at three months (mean difference 2.5 kg, 95 % CI −0.5 to 5.6) and at six months (mean difference 2.5 kg, 95 % CI −1.2 to 6.1). The mean values observed in the intervention group for mid arm circumference, mid arm muscle circumference, triceps skin fold thickness and right hand grip strength were greater than for the control group at all post hospital discharge time points. The economic evaluation suggested that it was feasible to collect resource use and EQ-5D data for a full cost-effectiveness analysis. Thematic analysis of 15 interviews identified three main themes related to the intervention and the trial: 1) a positive experience, 2) the reasons for taking part, and 3) uncertainty of the study process.

**Conclusions:**

This study demonstrated that home enteral feeding by jejunostomy was feasible, safe and acceptable to patients and their carers. Whether home enteral feeding as ’usual practice’ is a cost-effective therapy would require confirmation in an appropriately powered, multi-centre study.

**Trial registration:**

UK Clinical Research Network ID 12447 (main trial, first registered 30 May 2012); UK Clinical Research Network ID 13361 (qualitative substudy, first registered 30 May 2012); ClinicalTrials.gov NCT01870817 (first registered 28 May 2013)

## Background

Weight loss and poor nutritional intake is frequently observed after oesophagectomy for cancer.^.^ Ryan et al. [1] estimated that at hospital discharge after oesophagectomy, oral intake was sufficient to meet only 65 % of estimated energy requirements. A recent review identified between 5 % and 12 % weight loss at six months after surgery with more than half of patients losing more than 10 % of preoperative weight [2]. In the studies to report long-term outcome [3, 4], at five years or more after surgery, between 49 % and 95 % of patients had failed to regain lost weight postoperatively. This suggests that the early deleterious effects on weight and nutrition have a sustained and durable effect. Whether better weight maintenance in the early months translates into long-term benefit is unknown.

Several centres have reported on the value of home enteral feeding in selected patients after oesophagectomy [5–11]. Feeding in this setting has largely been restricted to patients with early nutritional failure or anastomotic complications that mandate an extended period of no oral intake. The practice of routine home enteral feeding after oesophagectomy has not been established.

The frequency of jejunostomy placement at the time of oesophagectomy varies widely between centres [12]. Within the UK, some centres place a jejunostomy in fewer than a quarter of patients undergoing resection, while other centres place a jejunostomy in over three quarters of patients. The 2010 UK National Oesophagogastric Cancer Audit identified that overall 68 % of the 2,200 patients undergoing oesophagectomy during the time period of October 2007 to June 2009 had a feeding jejunostomy placed [12]. Twenty-eight percent had no feeding adjunct and the remaining 4 % of patients had an alternative adjunct, such as a nasojejunal tube. In addition to heterogeneity in the practice of tube placement, there are variations in the feeding infusion regimens, with each centre adopting its own local policy.

It is possible that the observed heterogeneity in practice stems from the rare but serious in-hospital complications associated with jejunostomy tube placement and feeding. A review of published series indicates a revision laparotomy rate of 0.9 % (range 0 to 3 %) [13]. These relate to small bowel necrosis, perforation, obstruction or feed tube migration into the peritoneal cavity. None occurred after hospital discharge, suggesting that it is safe to discharge patients home with the tube in place.

The physical, psychological and emotional consequences of living with a feeding jejunostomy tube and the associated feeding are unknown, from both the patient and carer perspectives. Studies of patients receiving home feeding via gastrostomy tubes suggest that the tube itself and the associated regimen may impose a burden of treatment [14–16].

The objective of this study was to pilot an investigation of the impact of six weeks of home jejunostomy feeding in patients undergoing oesophagectomy or total gastrectomy for cancer, and therefore to assess the feasibility of conducting a subsequent appropriately powered multi-centre trial.

## Methods

The study protocol has previously been published in full [17]. A summary is provided below.

### Study design

This was a prospective two-arm randomised controlled pilot and feasibility trial, with a nested qualitative study, comparing six weeks of home jejunostomy feeding (intervention) with treatment as usual (control). Given the nature of the intervention and control, it was not possible to blind participants or those responsible for patient care. To minimise bias, the research dietitians (MB, VH) who collected study data had no involvement in patient care.

### Participants and setting

The study recruited adult patients referred to the University Hospitals of Leicester NHS Trust Oesophago-gastric Cancer Service, Leicester, United Kingdom with confirmed diagnoses of oesophageal or gastric cancer. Inclusion criteria were planned elective oesophagectomy (transhiatal, Ivor Lewis, three-stage) or total gastrectomy with placement of feeding jejunostomy tube. Patients undergoing subtotal gastrectomy were excluded, as it is not our usual practice to place feeding jejunostomy tubes in this patient group.

### Recruitment and ethics

Potential participants were identified at the weekly multidisciplinary, upper gastro-intestinal cancer meetings by a team member involved in the patient’s care. At the surgical clinic visit, potential participants were asked by a member of the healthcare team whether they were happy to receive information about the study. If they agreed, they were provided with a participant information leaflet and asked to give their consent for a member of the research team to contact them by telephone.

A minimum of 24 hours later a member of the research team contacted the potential participant by telephone. If they agreed to take part, the patient was visited in hospital at their pre-assessment clinic appointment. Written informed consent was obtained from all trial participants.

Research Ethics Committee approval for the study was granted by the Nottingham Local Research Ethics Committee 2, Nottingham, NG1 6FS (protocol 11/EM/0383) in January 2012. Recruitment commenced in July 2012 and closed in March 2014. Participant follow-up was completed in September 2014.

### Randomisation

Participants were randomised to control or intervention group at enrolment, and prior to surgery, so that baseline quality of life and nutritional parameters could be collected. The randomisation schedule was managed by the University of Leicester Clinical Trials Unit (hosted by Sealed Envelope Ltd), using computer-generated random assignment, using permuted blocks, stratified for type of procedure (oesophagectomy or total gastrectomy). A member of the Research Team (Dietitian or Lead Clinician) randomised the participant through an electronic interactive web response system (IWRS). Participants were entered into the study sequentially, and the IWRS provided a trial participation number.

### Standard postoperative care

All participants received standard postoperative care while in hospital, consisting of feeds, via the jejunostomy tube, placed at time of surgery. Tube insertion, commencement of feeds and subsequent increase in volume followed a previously agreed care pathway [17]. Continuous jejunostomy feeds were reduced to supplementary overnight feeds (10 – 15 hours duration) when oral intake recommenced after surgery (at approximately post operative day 7). Overnight feed continued until the morning of the day of hospital discharge in all participants. Dietary advice, including food fortification and the use of prescribable nutritional supplements, with supporting written information, was provided to all patients, prior to discharge, by the clinical team.

### Intervention

Participants randomised to the intervention arm were referred to the local Home Enteral Nutrition Service and taught (with or without carer support) to independently manage the jejunostomy feed at home. The intention was to administer overnight jejunostomy feeds via an electronic pump for the first 6 weeks after discharge from hospital. The goal of supplementary jejunostomy feeding was to provide at least 50 % of energy and protein requirements.

### Control

Participants randomised to the control group received routine clinical care. This comprised discontinuation of jejunostomy feeds on the day of hospital discharge. The tube was left in situ until outpatient review at week six after discharge. As per usual practice, home jejunostomy feeds were recommenced when deemed necessary by the clinical team or Home Enteral Nutrition dietitian. The criteria for recommencing feed comprised weight loss of greater than 5 % from baseline level, reduced functional status or estimated oral calorie intake <33 % of requirements.

### Outcome measures

The primary outcome measures were recruitment rate to the study and retention rates at six weeks and six months post baseline. These measures were selected to determine whether or not an appropriately powered definitive trial would be possible.

The secondary outcome measures, recorded at hospital discharge, six weeks after discharge, three months after surgery and six months after surgery, focused on nutritional status and quality of life, specifically:

i. the nutritional parameters of weight, body mass index, upper arm anthropometry, and grip strength
ii. nutritional intake including total energy (kcal/day) and protein intake (g/day), contribution of oral intake (food, fluids), oral nutritional supplements and jejunostomy feed (to be reported elsewhere)
iii. generic (EORTC QLQ-C30 [18]) and disease-specific (EORTC QLQ-OG25 [19]) quality of life measures, in order to assess the variability of these measures and the relationship between generic and disease-specific measures
iv. cost-effectiveness, derived from the EQ-5D-3 L [20] quality of life instrument (measured at three and six months post baseline) and the healthcare costs for the duration of the study period
v. jejunostomy tube complications
vi. hospital readmission rates
vii. Participants’ and their carers’ experiences of living with a feeding jejunostomy tube and home feeding.

### Data analysis

Key elements of the data analysis plan have been reported previously [17].

The proportion of eligible patients who consented to participate were calculated, along with the proportions in each intervention group completing six weeks and six months of assessments. Hospital readmission rates were summarised by intervention group.

The analysis was performed on the full analysis set, analysing according to group assigned by randomisation. The full analysis set comprised all patients who were randomised to one of the trial interventions and who had post randomisation endpoint data recorded, regardless of the actual intervention the patient received, and regardless of protocol deviations or completion of the trial. Additionally, some sensitivity analyses were performed in which patient groups were analysed according to whether or not they received the jejunostomy feeding while being randomised to intervention or control. As the likely outcome measure for the multi-centre trial will be quality of life, point estimates and variability of each of the quality of life measures were determined by intervention group (assuming either different or same standard deviations). As this was a pilot and feasibility study, it was not powered to detect differences between the two groups. Accordingly, formal hypothesis testing has not been conducted.

The differences in EQ-5D-3 L, EORTC QLQ-C30 and EORTC QLQ-OG25 between the groups, with associated 95 % CI, were calculated at three weeks after hospital discharge, three months after surgery and six months after surgery, based on ANCOVA analyses to adjust for type of surgery and baseline value of EORTC QLQ-C30. The EQ-5D-3 L was summarised using the UK time trade-off (TTO) value set. For OG25, the summary outcome of all questions was determined, while for C30 the focus was on the quality of life questions.

### Sample size

As this was a feasibility study, the sample size was selected in order to enable a sensible estimation of the quantities of interest, in particular variability, while not exposing too large a number of participants to the full range of experimental procedures. The intention was to recruit 60 participants, 30 randomised to receive home jejunostomy feeding and 30 to receive treatment as usual. This made allowance for a 17 % early withdrawal rate, which would result in 50 participants completing the six-week intervention period.

### Qualitative study

Potential participants for the qualitative exploratory study were those recruited into the main trial, and their carers. Purposive sampling was undertaken in order to ensure a representative selection of cases, stratifying for gender, age, cohabiting status and treatment group. Face-to-face semi-structured interviews were conducted by one of the researchers (VH), with the aim of exploring the experiences of people with oesophago-gastric cancer and their carers regarding living with a jejunostomy tube. More specifically relating to the feasibility of the randomised controlled trial, participants were asked to describe their experiences of taking part in the study. Interviews lasted between 21 and 75 minutes; they were audio-recorded and then transcribed verbatim. Following familiarisation with the data, an inductive thematic analysis was conducted with cross-checking of themes between two members of the research team (MB and VH). Field notes made following each interview were also taken into consideration during the analysis stage.

## Results

Over the 20-month recruitment period, 112 eligible patients were screened for inclusion in the study. Fifty-four agreed to participate, a recruitment rate of 48 %. This represented 90 % of the projected sample size. Twenty-six participants were randomised to the intervention arm, while 28 participants were randomised to the control arm. Baseline and pre-intervention characteristics are summarised in Table 1.

**Table 1:** Participant characteristics

	Intervention (*n* = 20)	Control (*n* = 21)
Sex		
Male	18 (90 %)	18 (86 %)
Female	2 (10 %)	3 (14 %)
Age in years	64.6 (8.0)	63.1 (8.7)
Body mass index at baseline (kg/m^2^)	27.0 (4.9)	28.4 (4.2)
Percentage weight loss from diagnosis to visit 1	−0.8 (10.5)	−1.2 (9.2)
Tumour location		
Lower third oesophagus	13 (65 %)	14 (67 %)
Cardia	6 (30 %)	5 (24 %)
Linitis plastica	1 (5 %)	2 (10 %)
Neoadjuvant chemotherapy	20 (100 %)	18 (86 %)
UICC stage		
0	0 (0 %)	1 (5 %)
1	3 (15 %)	2 (10 %)
2	3 (15 %)	9 (43 %)
3	13 (65 %)	9 (43 %)
4	1 (5 %)	0 (0 %)
T stage		
Tis	0 (0 %)	1 (5 %)
T1	0 (0 %)	0 (0 %)
T2	4 (20 %)	4 (19 %)
T3	15 (75 %)	15 (71 %)
T4	1 (5 %)	1 (5 %)
N stage		
N0	5 (25 %)	7 (33 %)
N1	8 (40 %)	12 (57 %)
N2	7 (35 %)	2 (10 %)
N3	0 (0 %)	0 (0 %)
Type of surgery		
Transhiatal oesophagectomy	2 (10 %)	1 (5 %)
Ivor Lewis oesophago-gastrectomy	14 (70 %)	15 (71 %)
Total gastrectomy	4 (20 %)	5 (24 %)
Surgical approach		
^a^Open	8 (40 %)	8 (38 %)
Laparoscopic	12 (60 %)	13 (62 %)
ICU/HDU stay in days	6.2 (3.6)	6.0 (4.6)
Hospital stay in days	19.4 (6.7)	16.3 (6.8)

Values indicated are mean (standard deviation) for continuous measures, and counts (percentages) for categorical measures

*HDU* high dependency unit, *ICU* intensive care unit, *SD* standard deviation, *Tis* in situ carcinoma, *UICC* Union for International Cancer Control

^a^All total gastrectomy and transhiatal oesophagectomy procedures were performed through open access. Ivor Lewis oesophago-gastrectomy procedures were performed through laparoscopic abdominal and open thoracic access. Radiotherapy, either preoperative or postoperative, was not employed in any participant

**Fig. 1:**
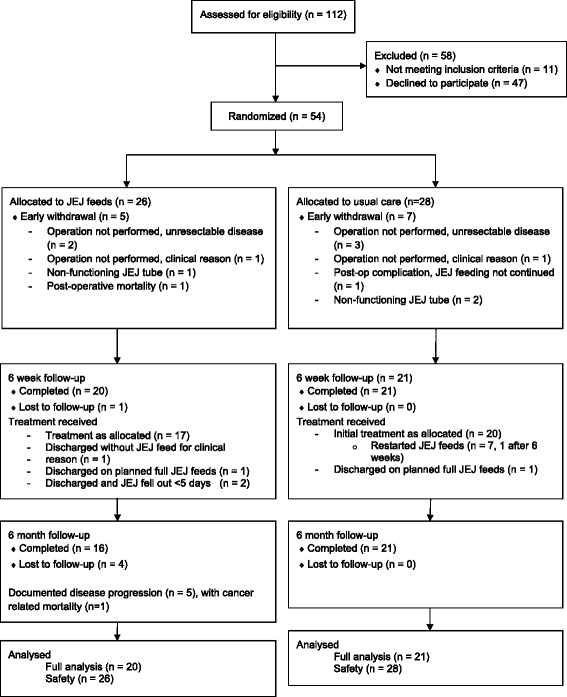
CONSORT diagram indicating participant disposition

Figure 1 shows the disposition of patients throughout the study. Study retention at six weeks was 41 participants (41/54, 76 %); it was 36 of 54 participants (67 %) at six months. Seven of the 21 participants (33 %) in the control group required home enteral feeding during the first six weeks after discharge. One was discharged from hospital on planned feeding because of a small anastomotic leak managed non-operatively. Six participants recommenced home enteral feed on the direction of the clinical team, because of greater than 5 % weight loss from baseline or hospital discharge value and deteriorating functional status. Sixteen participants, eight in the intervention group (total home feeding duration: 54–172 days) and eight in the control group (total home feeding duration: 70–133 days). All had discontinued feeding by six months.

Tables 2 and 3 show the EORTC QLQ-C30 and QLQ-OG25 quality of life scores for the two groups. The global quality of life scores deteriorated in both groups after surgery, but approached baseline levels in both groups by six months. A similar pattern is noted in the disease-specific QLQ-OG25 scores. Estimates of the standard deviation for the QLQ-C30 scores were 23, 22 and 21 at six weeks after hospital discharge, three months after surgery and six months after surgery respectively. Similar values for EORTC QLQ-OG25 scores were 15, 15 and 14 respectively.

**Table 2:** EORTC QLQ-C30 quality of life scores

	Absolute baseline QOL scores		Six weeks after hospital discharge (change)		Three months after surgery (change)		Six months after surgery (change)	
	Int (*n* = 20)	Cont (*n* = 21)	Int (*n* = 20)	Cont (*n* = 21)	Int (*n* = 18)	Cont (*n* = 20)	Int (*n* = 16)	Cont (*n* = 20)
Global QOL	74 (22)	80 (17)	−20 (37)	−21 (16)	−17 (29)	−21 (22)	−5 (25)	−7 (18)
Physical	92 (10)	93 (15)	−27 (20)	−21 (20)	−22 (21)	−17 (21)	−17 (21)	−9 (21)
Role	85 (23)	90 (22)	−47 (31)	−41 (28)	−43 (31)	−36 (28)	−17 (31)	−19 (37)
Cognitive	84 (20)	91 (16)	−9 (28)	−6 (17)	−5 (28)	−2 (18)	−13 (25)	−6 (16)
Emotional	77 (22)	83 (20)	−2 (17)	−4 (19)	−1 (15)	−7 (15)	1 (19)	−3 (16)
Social	87 (24)	86 (24)	−34 (39)	−34 (29)	−34 (42)	−24 (29)	−13 (30)	−15 (33)
Fatigue	13 (14)	17 (29)	37 (26)	28 (25)	34 (32)	27 (23)	27 (27)	16 (28)
Nausea & vomiting	9 (21)	8 (23)	19 (42)	10 (31)	19 (39)	19 (35)	13 (28)	10 (38)
Pain	4 (12)	13 (21)	24 (19)	9 (29)	18 (22)	7 (28)	15 (21)	1 (24)
Dyspnoea	3 (10)	3 (10)	33 (31)	19 (27)	31 (35)	22 (22)	23 (23)	10 (22)
Sleep disturbance	20 (29)	13 (20)	25 (42)	24 (32)	13 (43)	12 (25)	2 (43)	7 (32)
Appetite loss	13 (29)	19 (37)	57 (46)	32 (39)	48 (50)	25 (36)	17 (37)	13 (48)
Constipation	10 (19)	17 (34)	−5 (16)	−2 (41)	7 (27)	8 (43)	0 (21)	−3 (40)
Diarrhoea	0 (0)	5 (12)	13 (20)	21 (29)	15 (21)	22 (27)	25 (26)	15 (20)
Financial impact	27 (40)	19 (31)	−3 (34)	9 (26)	−4 (32)	10 (27)	−6 (37)	7 (30)

Values indicated are mean (standard deviation)

*Cont* control, *Int* intervention, *QOL* quality of life

**Table 3:** EORTC QLQ-OG25 quality of life scores

	Absolute baseline QOL scores		Six weeks after hospital discharge (change)		Three months after surgery (change)		Six months after surgery (change)	
	Int (*n* = 20)	Cont (*n* = 21)	Int (*n* = 20)	Cont (*n* = 21)	Int (*n* = 18)	Cont (*n* = 20)	Int (*n* = 16)	Cont (*n* = 20)
Body image	87 (23)	95 (16)	−8 (32)	−5 (24)	−2 (33)	−12 (33)	0 (24)	−13 (29)
Dysphagia	82 (28)	84 (27)	−3 (47)	0 (35)	4 (37)	4 (36)	12 (29)	7 (33)
Eating	73 (35)	72 (32)	−24 (50)	−6 (39)	−12 (44)	−3 (37)	−2 (36)	3 (42)
Reflux	88 (27)	94 (11)	−15 (39)	−17 (30)	−9 (35)	−12 (22)	−2 (12)	−13 (27)
Odynophagia	84 (27)	79 (28)	−7 (39)	7 (32)	−3 (40)	7 (38)	0 (21)	8 (32)
Pain & discomfort	93 (13)	92 (17)	−23 (22)	−12 (20)	−15 (28)	−17 (32)	−19 (27)	−11 (28)
Anxiety	39 (20)	51 (30)	8 (31)	17 (34)	18 (28)	8 (24)	25 (30)	18 (35)
Eating with others	80 (37)	89 (27)	8 (43)	3 (21)	7 (42)	2 (30)	10 (38)	3 (37)
Dry mouth	67 (34)	71 (34)	−15 (52)	2 (34)	−7 (44)	−2 (40)	0 (37)	8 (36)
Trouble with taste	82 (33)	76 (35)	−33 (41)	−16 (42)	−20 (43)	−12 (38)	−17 (49)	−8 (49)
Trouble swallowing saliva	88 (27)	92 (23)	3 (30)	5 (26)	11 (28)	7 (26)	8 (29)	3 (28)
Choked when swallowing	90 (19)	97 (10)	0 (31)	0 (15)	6 (17)	−2 (13)	4 (17)	−2 (7)
Trouble with coughing	75 (26)	79 (25)	−28 (39)	−13 (27)	−24 (49)	−7 (32)	−15 (37)	−5 (33)
Trouble talking	95 (16)	92 (18)	−7 (28)	2 (17)	−7 (31)	2 (17)	4 (17)	0 (31)
Weight loss	88 (22)	89 (19)	−17 (45)	−9 (24)	−22 (44)	−18 (23)	−19 (37)	−8 (30)
Hair loss	81 (21)	85 (34)	6 (25)	−17 (28)	−7 (15)	13 (38)	0 (0)	−17 (24)

Values indicated are mean (standard deviation)

*Cont* control, *Int* intervention, *QOL* quality of life

Table 4 summarises the nutritional measures for the two groups. At six weeks, participants in the control group had lost on average 3.9 kg more than participants in the intervention group (95 % CI 1.6 to 6.2). These differences remained evident at three months (mean difference 2.5 kg, 95 % CI −0.5 to 5.6) and at six months (mean difference 2.5 kg, 95 % CI −1.2 to 6.1). At six weeks, the mean difference in BMI between the control and intervention groups was 1.3 kg/m^2^ (95 % CI 0.6 to 2.1).

**Table 4:** Change in nutritional characteristics from baseline

	Six weeks after hospital discharge		Three months after surgery		Six months after surgery	
	Intervention (*n* = 20)	Control (*n* = 21)	Intervention (*n* = 18)	Control (*n* = 21)	Intervention (*n* = 16)	Control (*n* = 21)
Weight (kg)	−3.8 (3.5)	−8.6 (4.7)	−6.3 (5.1)	−9.7 (5.7)	−7.4 (5.2)	−10.9 (7.2)
Weight (%)	−4.6 (3.9)	−9.7 (4.8)	−7.4 (5.7)	−10.9 (6.0)	−8.9 (5.4)	−12.2 (7)
Weight loss experienced from baseline						
None	1 (5 %)	1 (5 %)	1 (6 %)	1 (5 %)	1 (6 %)	0 (0 %)
<5 %	12 (60 %)	2 (10 %)	8 (44 %)	2 (10 %)	3 (19 %)	3 (14 %)
5% to 10 %	5 (25 %)	8 (38 %)	3 (17 %)	5 (24 %)	4 (25 %)	4 (19 %)
>10 %	2 (10 %)	10 (48 %)	6 (33 %)	13 (62 %)	8 (50 %)	14 (67 %)
Mid arm circumference (cm)	−2.1 (1.4)	−2.8 (1.9)	−2.4 (1.6)	−2.9 (1.7)	−2.6 (1.6)	−3.1 (2.3)
Mid arm circumference (%)	−6.2 (4.0)	−8.4 (5.4)	−6.9 (4.5)	−8.7 (4.9)	−7.6 (4.6)	−9.1 (6.3)
Mid arm muscle circumference (cm)	−2.1 (1.4)	−2.8 (2.1)	−2.4 (1.6)	−2.9 (1.8)	−2.6 (1.5)	−3.1 (2.3)
Mid arm muscle circumference (%)	−6.2 (4.8)	−8.4 (7.1)	−6.9 (5.5)	−8.7 (6.1)	−7.6 (4.9)	−9.1 (7.4)
Triceps skin fold thickness (mm)	−0.7 (2.1)	−1.8 (2.2)	−1.1 (2.3)	−2.3 (2.3)	−1.9 (2.7)	−2.5 (2.7)
Triceps skin fold thickness (%)	−2.7 (11.8)	−8.2 (13.5)	−2.7 (15.7)	−12.0 (14.0)	−8.7 (16.2)	−11.6 (13.6)
Hand grip dynamometry (kg)	−2.5 (4.4)	−4.1 (4.9)	−2.9 (4.6)	−4.1 (4.3)	−1.5 (4.4)	−2.0 (4.1)
Hand grip dynamometry (%)	−6.9 (12.6)	−11.4 (12.7)	−7.8 (14.5)	−11.9 (11.3)	−3.5 (12.7)	−5.2 (10.4)

Values indicated are mean (standard deviation)

The mean values observed in the intervention group for mid arm circumference, mid arm muscle circumference, triceps skin fold thickness and right hand grip strength were greater than for the control group at all post hospital discharge time points (Table 4).

The hospital readmission rates during the first six weeks after discharge from hospital were 6 of 20 participants (30 %) in the intervention group and 5 of 21 participants (24 %) in the control group. The corresponding figures for the time period between six weeks after discharge and three months postoperatively were 2 of 18 (11 %) for the intervention group and 4 of 21 (19 %) for the control group. During the last three months of the study period, the readmission rates were 1 of 16 (6 %) for the intervention group and 2 of 21 (9 %) for the control group.

Table 5 summarises the observed jejunostomy complications, both in hospital and out of hospital. The only two major (Clavien-Dindo grade 3b or greater) jejunostomy tube- or feed-related complications occurred in hospital, prior to commencement of the home feeding schedule [21]. These two participants in the control arm, who had undergone total gastrectomy, required laparotomy and small bowel resection for feed-related small bowel necrosis. There were no major (Clavien-Dindo grade 3b or greater) tube- or feed-related complications after discharge from hospital [21]. Table 6 summarises the in-hospital outcome (prior to commencement of the intervention period).

**Table 5:** Comparison of ^1^minor jejunostomy complications for the two groups

Minor jejunostomy complications	^2^Intervention (*n* = 22)	^3^Control (*n* = 23)
In hospital		
Any jejunostomy complication (%)	11 (50 %)	7 (30 %)
Diarrhoea (%)	2 (9 %)	3 (13 %)
Reflux of feed/vomiting (%)	0 (0 %)	0 (0 %)
Tube displacement or migration (%)	0 (0 %)	1 (4 %)
Inadvertent tube removal (%)	1 (4 %)	1 (4 %)
Tube fracture (%)	0 (0 %)	0 (0 %)
Leakage around insertion site (%)	5 (23 %)	1 (4 %)
Tube occlusion (%)	4 (18 %)	3 (13 %)
^2^Functional jejunostomy at hospital discharge	20	21
Out of hospital	Intervention (*n* = 20)	Control (*n* = 21)
Any jejunostomy complication (%)	11 (55 %)	14 (67 %)
Diarrhoea (%)	4 (20 %)	3 (14 %)
Reflux of feed/vomiting (%)	2 (10 %)	0 (0 %)
Tube displacement or migration (%)	1 (5 %)	0 (0 %)
Inadvertent tube removal (%)	3 (15 %)	5 (24 %)
Tube fracture (%)	0 (0 %)	0 (0 %)
Leakage around insertion site (%)	4 (20 %)	4 (19 %)
Tube occlusion (%)	2 (10 %)	2 (9 %)
Functional jejunostomy at end of six weeks (%)	16 (80 %)	16 (76 %)

^1^Indicates Clavien-Dindo grade 1 or 2 complications

^2^One participant had a non-functioning jejunostomy tube and one participant underwent gastric mobilisation with jejunostomy placement, but did not proceed to resection

^3^One participant had a non-functioning jejunostomy tube, and for one participant the jejunostomy fell out

**Table 6:** Comparison of in-hospital course for the two groups

	Intervention (*n* = 22)	Control (*n* = 23)
Abdominal sepsis		
Radiological drainage (%)	0 (0 %)	0 (0 %)
Other non-operative (%)	0 (0 %)	0 (0 %)
Return to operating theatre (%)	1 (4.5 %)	2 (9 %)
Anastomotic leak		
Radiological drainage (%)	0 (0 %)	2 (9 %)
Other non-operative (%)	1 (4.5 %)	3 (13 %)
Return to operating theatre (%)	2 (9 %)	1 (4 %)
Cardiac complication		
Arrhythmia (%)	4 (18 %)	2 (9 %)
Cardiac failure (%)	0 (0 %)	0 (0 %)
Chylothorax		
Non-operative (%)	1 (4.5 %)	0 (0 %)
Return to operating theatre (%)	2 (9 %)	0 (0 %)
Haemorrhage		
Blood transfusion (%)	1 (4.5 %)	0 (0 %)
Return to operating theatre (%)	1 (4.5 %)	0 (0 %)
Pneumonia/pleural effusion		
Intercostal drain placement (%)	3 (13 %)	3 (13 %)
Re-ventilation (%)	2 (9 %)	4 (17 %)
Renal complication (%)	0 (0 %)	0 (0 %)
Surgical site infection		
Superficial infection (%)	5 (23 %)	2 (8 %)
Deep infection (%)	2 (9 %)	4 (17 %)
Thromboembolic disease		
Deep venous thrombosis (%)	1 (4.5 %)	0 (0 %)
Pulmonary embolism (%)	0 (0 %)	0 (0 %)
In-hospital mortality (%)	1 (4.5 %)	0 (0 %)

### Economic evaluation

The generic health-related quality of life was measured using EQ-5D-3 L. The mean EQ-5D score was slightly lower in the intervention group than in the control group (Table 7).

**Table 7:** Cost per patient by group and EQ-5D score

Cost per patient	Intervention	Standard care	Mean difference
Baseline	£ 2,451.58 (3,535.33)	£ 1,669.57 (2,004.62)	£ 782.01
Six weeks	£ 2,438.00 (2,440.52)	£ 1,795.74 (2,422.75)	£ 642.26
Six months	£ 1,012.49 (1042.42)	£ 1,723.61 (2,681.00)	£ -711.12
Total cost	£ 3,450.49	£ 3,519.35	£ -68.86
EQ-5D score	Intervention	Standard care	Mean difference
Baseline	0.800 (0.181)	0.825 (0.202)	−0.025
Six weeks	0.599 (0.239)	0.643 (0.255)	−0.044
Six months	0.686 (0.201)	0.735 (0.274)	−0.049

Values indicated are mean (standard deviation)

Complete data on resource use were available for 45 participants at baseline (24 control, 21 intervention), 41 participants at six weeks after discharge from hospital (21 control, 20 intervention) and 36 participants at six months after surgery (21 control, 15 intervention), suggesting that data collection using cost questionnaires was feasible.

The mean cost per patient in the intervention arm was £ 3450 and in the control arm was £3,519 (Table 7). This difference was largely accounted for by an in-patient stay of 16 days for one patient in the control arm six weeks after discharge.

A mapping technique was used to link the outcomes from EORTC QLQ-C30 onto the EQ-5D to inform whether quality-adjusted life years (QALYs) could be worked out from the EORTC QLQ-C30 for future study. The results showed that the algorithm by McKenzie and van der Pol [20], using the OLS model, did not accurately predict the EQ-5D values.

### Qualitative study findings

Fifteen interviews were conducted, between two and three months post surgery. Twelve of the participants were male and three female with eight interviews also including a carer or partner, all of whom were female. The mean age of the patients was 65 years (range 52 to 74 years). Ten of the 15 patient participants were married or cohabiting. All participants had a jejunostomy tube placed at the time of surgery that was then used in the immediate postoperative course. Eleven of the 15 subsequently received home jejunostomy feeding, the duration of which varied between 28 and 104 days.

Regarding the feasibility of conducting a randomised controlled trial involving home jejunostomy feeding, the interviews revealed three main themes: 1) a positive experience (15 participants), 2) the reasons for taking part (11 participants), and 3) uncertainty of the study process (9 participants). All participants described coping mechanisms for managing the feeding tube and high levels of compliance with jejunostomy tube care and the feeding regimen. The in-depth experiences of the challenges and motivators of living with a jejunostomy tube, and in particular the coping strategies that were described by patients and their carers, have been reported elsewhere [22].

The general consensus from all of the participants was that it was ‘not a problem taking part’ in the study. Interestingly, most patients (*n* = 8) voiced that they were pleased to be in the group to which they were randomised.

*“Fortunately, for me, anyway, I was one of those that didn’t have to [be fed]…… Beforehand, it didn’t really mean an awful lot, but I was relieved afterwards, yeah, when I found that out.”*

*“I wouldn’t have liked that [being in the control group], I would have thought that that was wasting my time, so I’m glad that I’m in the one …… I think that just feeding people for 6 weeks like that has got to be a good thing. It takes all the worry out of it for both staff and patients and it’s no big deal.”*

None of the participants found the additional visits by the research team to be burdensome. Only one negative comment was made regarding the assessment tools. This related to the difficulty in completing the quality of life questionnaire and the dilemma of feeling ‘well’ but knowing that you have cancer. Most of the participants (*n* = 11) spoke about why they had taken part in the study. In some cases (*n* = 8) this was for altruistic reasons as well as the hope that it would improve their wellbeing.

*“I thought well, you know, if it benefits people, well even if it benefits me, which I think it has done, and if it benefits people in the long term I think it’s a good thing.”*

For one patient it was because she thought that it would mean that she received better care.

*“Well I was hoping I was going to be in it because me son said they take more notice of you, take more care of you.”*

Finally, there were a number of comments from participants (*n* = 9) that suggested that despite having the information sheet and giving consent, they did not fully understand the study process.

*“I wonder, this may not be true at all , but I did wonder it passed through my head when I knew I was in this particular group……was I put in to this group actually because I made such a good recovery?”*

*“I just came home…. and then all of a sudden the research dietitian phones up and says ‘when are you coming in?’ and I said ‘what for?’ she said ‘for this study’. I said ‘oh, am I on it then?’”*

## Discussion

The aim of most previous studies of jejunostomy feeding after oesophago-gastric resection has been to determine whether preoperative or in-hospital feeding would influence in-hospital outcome measures, such as postoperative complications or length of hospital stay [23–26]. These studies reported either no benefit or marginal benefit in favour of in-hospital feeding. The current study is the first randomised trial to compare a planned programme of out-of-hospital enteral feeding to usual care. The principal findings were that home feeding was safe, acceptable to patients and their carers, and that it might confer nutritional benefits. Ninety percent of the projected 60 participants were recruited to the trial.

The relatively high non-completion rate of 33 % was considerably greater than the projected 17 % [17], but largely relates to participant enrolment before surgery. In the planning of this study, the timing of enrolment was one of the key considerations and discussion points. It was agreed that recruitment should be before surgery for several reasons. Firstly, this allowed baseline pre-surgery information to be collected. Secondly, it prevented the study team from biasing who would take part based on postoperative course. Thirdly, the study management group, which included patient and carer representatives, considered that patients convalescing after surgery would not be well placed to deal with the information-giving exercise and consenting for enrolment in a clinical study. It was considered that this might adversely affect recruitment and acceptability. The recommendations for a definitive trial would be that recruitment be likewise before surgery.

The other finding that was not anticipated was the relatively high requirement for recommencement of home feeding in the control group, at 33 %. The study was set up, anticipating that 15–20 % of participants in the control arm would require ’rescue‘ feeding [17]. One possible explanation for this observation is the high level of dietetic support offered to patients at our centre. It may be that the threshold for restarting home feeding as ’usual treatment‘ is lower than at other centres, although pre-defined criteria were used as triggers for restarting feeding.

The demographics of the sample were as expected, with a male preponderance and a mean age around 65 years. In the study, the groups were stratified for type of surgery (oesophagectomy versus total gastrectomy) because of concerns about the differing effect of the two operations on weight loss and quality of life. No direct comparison of the outcomes of participants undergoing oesophagectomy has been made with those of participants undergoing total gastrectomy. The rationale for stratifying the trial based on type of surgery was on the premise that the type of reconstruction after total gastrectomy might have an independent effect on weight and quality of life. For a definitive trial, we consider that such stratification would be required. The alternative approach would be to enter a homogeneous group into a larger scale study. This could be achieved by restricting the inclusion criteria to patients undergoing Ivor Lewis style oesophago-gastrectomy. The rationale for enrolling patients undergoing total gastrectomy in the current study was to make the findings as generalisable as possible, and also because this reflected our local practice of placing feeding jejunostomy in patients undergoing these operations.

During the study, one change was made to the protocol. The window of assessments for visit 4 was initially set at 48 hours, but this proved impractical for patients recruited around the time of public holidays, and this time window was increased to 96 hours. Going forward, it might prove easier to reference every assessment point relative to one fixed point in time. In this study, two reference points were used: day of surgery and day of hospital discharge. This meant that the interval between visit 3 (six weeks after hospital discharge) and visit 4 (three months after surgery) varied considerably between participants. The initial intention had assumed a postoperative hospital stay of two weeks. In those with a longer hospital stay, the interval between these two visits was short.

Although it is gaining acceptance, there are comparatively few reports in the literature relating to home jejunostomy feeding. There are wide variations in practice between centres. Several studies have reported on the use of home feeding on a selective basis. The indications for feeding have been either the management of postoperative complications, such as anastomotic leak where a prolonged period of no oral intake was required, or in those with nutritional failure in the early months after surgery. Ryan et al*.* [1] noted that 8 % of 205 patients who had undergone oesophagectomy were discharged from hospital on a planned programme of enteral feeding, and that a further 6 % had restarted feeding within the first month. Haverkort et al*.* [27] reported that 48 % of 80 patients after oesophagectomy were discharged home on planned feeding. By six months after surgery, this figure had reduced to 2 % and by 12 months, a further drop to 1 % was seen. Couper [6] reported that 19 % of 50 patients after oesophagectomy continued feeding after discharge, principally for poor oral intake and that a further 8 % restarted feeding later.

The use of planned rather than selective feeding has been reported in two small cohort studies. Tomascek et al*.* [9] reported on an enhanced recovery protocol that employed feeding exclusively via the jejunostomy for four weeks in order to facilitate early hospital discharge. Macharg et al*.* [11] demonstrated that home jejunostomy feeding was associated with a 2-kg absolute (relative 4 %) weight benefit compared to no supplementation.

The economic evaluation suggested that the home enteral nutrition cost the NHS slightly less than standard care. Although the cost difference was minimal, this needs further exploring in a future cost-effectiveness analysis, to ascertain whether the intervention is a cost neutral or cost saving option.

Findings from the qualitative study suggested that participants were happy to take part in this type of randomised controlled trial, and that the intervention and assessments were acceptable. Our findings around participants having incomplete understanding of the research process concur with those of previous work [28]. This highlights the importance of ensuring that researchers are confident that participants are able to give informed consent and fully understand, in particular, the randomisation process. Understanding of the reasons why patients agree to take part in a randomised controlled trial, which in this case, and as found previously [29], were mainly altruistic, may help optimise future study design.

The current study has allowed recruitment (48 %) and retention rates (76 % at six weeks, 67 % at six months) to be determined. The intention was for this information to inform a subsequent multi-centre, pragmatic randomised controlled trial. In planning the pilot study, it was envisaged that the primary endpoint for any subsequent definitive trial would be a generic quality of life measure, such as the QLQ-C30. Although not powered to detect a difference between the two groups, it remains unclear whether this remains an appropriate primary endpoint. In order to identify a target difference between the control and intervention groups of 10 points [30], such a trial would require 106 completing participants per group, assuming a 90 % power and a 5 % significance level. Allowing for 33 % early withdrawal and loss to follow-up rate would require recruitment of 161 participants per group. It may be that a measure of physical function is a more meaningful primary endpoint than quality of life.

This study has demonstrated safety and acceptability of home enteral feeding to patients and their caregivers. The measured benefits included better weight, muscle and fat store preservation. Quality of life measures were broadly similar between the two groups. The additional healthcare costs associated with home enteral feeding appeared to be offset by reduced healthcare costs in other areas.

## Conclusions

In conclusion, this study demonstrated that home enteral feeding by jejunostomy was feasible, safe and acceptable to patients and their carers. Whether home enteral feeding as ’usual practice‘ is a cost-effective therapy would require confirmation in an appropriately powered, multi-centre study.

## Abbreviations

ANCOVA: analysis of covariance
BMI: body mass index
CI: confidence interval
CONT: control
EORTC: European Organisation for Research and Treatment of Cancer
EQ-5D: quality of life tool used for health economics evaluation
HDU: high dependency unit
ICU: intensive care unit
INT: intervention
IWRS: interactive web response system
NHS: National Health Service
OG25: oesophago-gastric disease specific quality of life instrument
OLS: ordinary least squares
QALY: quality-adjusted life years
QLQ-C30: generic EORTC quality of life instrument
QOL: quality of life
TTO: time trade-off
UICC: Union for International Cancer Control
UK: United Kingdom
